# Cytotoxic Effects of Arsenite in Combination With Gamabufotalin Against Human Glioblastoma Cell Lines

**DOI:** 10.3389/fonc.2021.628914

**Published:** 2021-03-16

**Authors:** Bo Yuan, Kang Xu, Ryota Shimada, JingZhe Li, Hideki Hayashi, Mari Okazaki, Norio Takagi

**Affiliations:** ^1^ Laboratory of Pharmacology, Faculty of Pharmaceutical Sciences, Josai University, Sakado, Japan; ^2^ Department of Applied Biochemistry, Tokyo University of Pharmacy & Life Sciences, Hachioji, Japan; ^3^ Beijing Key Laboratory of Research of Chinese Medicine on Prevention and Treatment for Major Diseases, Experimental Research Center, China Academy of Chinese Medical Sciences, Beijing, China

**Keywords:** arsenite, gamabufotalin, glioblastoma, cell cycle arrest, p38 MAPK, autophagy, lactate dehydrogenase, combination therapy

## Abstract

Glioblastoma is a fatal primary malignant brain tumor, and the 5-year survival rate of treated glioblastoma patients still remains <5%. Considering the sustained development of metastasis, tumor recurrence, and drug resistance, there is an urgent need for the novel therapeutic approaches to combat glioblastoma. Trivalent arsenic derivative (arsenite, As^III^) with remarkable clinical efficacy in leukemia has been shown to exert cytocidal effect against glioblastoma cells. Gamabufotalin, an active bufadienolide compound, also shows selective cytocidal effect against glioblastoma cells, and has been suggested to serve as a promising adjuvant therapeutic agent to potentiate therapeutic effect of conventional anticancer drugs. In order to gain novel insight into therapeutic approaches against glioblastoma, the cytotoxicity of As^III^ and gamabufotalin was explored in the human glioblastoma cell lines U-87 and U-251. In comparison with U-251 cells, U-87 cells were highly susceptible to the two drugs, alone or in combination. More importantly, clinically achieved concentrations of As^III^ combined with gamabufotalin exhibited synergistic cytotoxicity against U-87 cells, whereas showed much less cytotoxicity to human normal peripheral blood mononuclear cells. G_2_/M cell cycle arrest was induced by each single drug, and further augmented by their combination in U-87 cells. Downregulation of the expression levels of cdc25C, Cyclin B1, cdc2, and survivin was observed in U-87 cells treated with the combined regimen and occurred in parallel with G_2_/M arrest. Concomitantly, lactate dehydrogenase leakage was also observed. Intriguingly, SB203580, a specific inhibitor of p38 MAPK, intensified the cytotoxicity of the combined regimen in U-87 cells, whereas wortmannin, a potent autophagy inhibitor, significantly rescued the cells. Collectively, G_2_/M arrest, necrosis and autophagy appeared to cooperatively contribute to the synergistic cytotoxicity of As^III^ and gamabufotalin. Given that p38 MAPK serves an essential role in promoting glioblastoma cell survival, developing a possible strategy composed of As^III^, gamabufotalin, and a p38 MAPK inhibitor may provide novel insight into approaches designed to combat glioblastoma.

## Introduction

Glioblastoma is one of the most common and lethal form of primary brain tumors, and characterized by fast infiltration, rapid growth, and resistance to conventional therapies (1, 2). Despite advances in the understanding of the nature of the molecular events associated with disease development and progression, the 5-year survival rate of treated glioblastoma still remains <5% (3). The median survival time for patients was only 14.6 to 20.9 months (4, 5). Considering the sustained development of metastasis, tumor recurrence, and drug resistance, there is an urgent need for the novel therapeutic approaches to combat glioblastoma.

It has been reported that trivalent arsenic derivatives (arsenite, As^III^) such as arsenic trioxide (As_2_O_3_) show superior therapeutic efficacy for acute promyelocytic leukemia (APL) patients (6, 7). These findings further opened the possibility of using As^III^ for other malignancies (8, 9). Several research groups including us have performed detailed systematic studies on the metabolites of As^III^ in APL patients (6, 7, 10, 11). We previously carried out a study of speciation of As_2_O_3_ in cerebrospinal fluid (CSF) samples from APL patients, and demonstrated for the first time that both inorganic arsenic and methylated metabolites existed in CSF, indicating that As^III^ is capable of penetrating into blood-brain barrier (10). In addition, As^III^ has been reported to show cytotoxicity toward glioblastoma cells through cell cycle arrest and autophagic cell death (12–14). These previous observations raise the possibility of utilizing As^III^ to treat patients with glioblastoma.

Natural products have been being received increased attention, due to their great potential in various cancer therapies. Bufadienolides are the major effective constituents of cinobufacini (also known as Huachansu), a well-known Chinese medicine that comes from the dried skin of *Bufo bufo gargarizans* Cantor, and cinobufacini has been employed to treat patients with different types of cancers such as hepatoma, gallbladder carcinoma, and lung cancer (15–17). We previously clarified that active bufadienolide compounds such as gamabufotalin and arenobufagin showed selective cytocidal effects against intractable cancer cells such as glioblastoma, but minimal effects on human normal peripheral blood mononuclear cells (PBMCs) (18) and mouse primary astrocytes (19). Notably, nearly non-toxic gamabufotalin concentrations on PBMCs effectively reduced the percentages of T-regulatory cells (Treg) cells (18), which has been characterized to play a critical role in limiting antitumor immune response and promoting immunological ignorance in cancer (20–22), suggesting the capability of gamabufotalin to enhance antitumor immunity. Additionally, bufadienolides have been demonstrated to enhance therapeutic efficacy of different types of cancer treatment (23, 24). These previous observations thus suggest that bufadienolides including gamabufotalin may serve as a promising adjuvant therapeutic agent to potentiate therapeutic effect of conventional anticancer drugs. However, whether gamabufotalin can sensitize glioblastoma cells to As^III^ has not yet been evaluated.

It has been demonstrated that cell cycle arrest, necrotic and autophagic cell death contribute to cytocidal effect of chemotherapeutic agents (19, 25–27). Cell cycle is coordinately and tightly regulated by the cyclin-dependent kinases (CDKs) and their associated regulatory cyclins (CDK/Cyclin complexes) (28, 29). Cdc25C has been demonstrated to play an important role in G_2_/M transitions of the cell cycle by activating cdc2/Cyclin B1 (28, 29). Survivin has been associated with increased malignancy of human gliomas, and considered to play vital roles in therapeutic resistance of primary glioblastoma cells (30). In addition, p38 kinase, a member of the mitogen-activated protein kinases (MAPKs) family, has been considered to be positively related to diverse cellular processes including cell cycle arrest and cell death signaling (31–33). Inversely, a novel prosurvival role of p38 MAPK has been demonstrated in human cancer cells such as glioblastoma cells (19, 34–36). Despite this, the molecular events underlying the potential cytotoxic effects caused by As^III^ and gamabufotalin, alone or in combination, against glioblastoma cells remain to be seen.

In the current study, in order to provide a novel insight into approach designed to combat glioblastoma, the cytotoxicity of As^III^ and gamabufotalin, alone or in combination, was investigated in the human glioblastoma cell lines U-87 and U-251 by focusing on proliferation inhibition associated with cell cycle arrest as well as cell death. PBMCs were also used to explore whether clinically achieved concentrations of As^III^ in combination with gamabufotalin show cytotoxic selectivity for cancer cells, rather than normal cells. Key regulatory molecules involved in cell cycle and cell death were investigated to further elucidate cytotoxic mechanisms. Whether p38 MAPK is implicated in As^III^ plus gamabufotalin-mediated cytocidal effects in U-87 cells was also investigated by using its specific inhibitor, SB203580.

## Materials and Methods

### Materials

Sodium arsenite (NaAsO_2_, As^III^) (>99% purity) and gamabufotalin (≥98% purity) were purchased from Tri Chemical Laboratories (Yamanashi, Japan) and Baoji Herbest Bio-Tech Co., Ltd. (Baoji, China), respectively. Wortmannin, a potent autophagy inhibitor, 2,3-bis(2-methoxy-4-nitro-5-sulfophenyl)-5-[(phenylamino)carbony]-2*H*-tetrazolium hydroxide (XTT), propidium iodide (PI), proteinase K, and ribonuclease A (RNaseA) were purchased from Merck KGaA (Sigma-Aldrich; Darmstadt, Germany). Dulbecco ***’*** s modified Eagle ***’*** s medium (DMEM) and phenazine methosulfate (PMS) were purchased from Wako Pure Chemical Industries (Osaka, Japan). Both SB203580, a specific inhibitor of p38 MAPK, and its negative control SB202474 were purchased from Merck KGaA. Fetal bovine serum (FBS) was obtained from Nichirei Biosciences (Tokyo, Japan). Can Get Signal ***^®^*** Immunoreaction Enhancer Solution was purchased from Toyobo Co., Ltd. (Osaka, Japan).

### Cell Culture and Treatment

Human glioblastoma cell lines U-87 and U-251 were obtained from the American Type Culture Collection (ATCC, Manassas, VA, USA) and cultured in DMEM supplemented with 10% heat-inactivated FBS and antibiotics [100 U/ml of penicillin and 100 μg/ml of streptomycin (Wako Pure Chemical Industries)] in a humidified 5% CO_2_ atmosphere at 37°C. Human peripheral blood mononuclear cells (PBMCs) were isolated from healthy volunteers using lymphocyte separation solution (d = 1.077) (Nacalai Tesque, Inc., Kyoto, Japan) according to the method previously described (18, 37). Briefly, 3 ml of heparinized blood was loaded on 3 ml of lymphocyte separation solution. After centrifugation at 400 × g for 30 min at room temperature, the opaque interface containing PBMCs was transferred to a clean centrifuge tube and washed three times with PBS. PBMCs were also cultured in RPMI-1640 medium supplemented with 10% heat-inactivated FBS, 100 U/ml of penicillin and 100 μg/ml of streptomycin in a humidified 5% CO_2_ atmosphere at 37°C. The cell density of PBMCs was adjusted to 5 × 10^5^ cells/ml prior to the treatments. This study has been approved by the IRB committee of Tokyo University of Pharmacy and Life Sciences. A written informed consent was obtained from all healthy volunteers. All methods were performed in accordance with the relevant guidelines and regulations. In experiments using inhibitors, U-87 cells were treated with respective inhibitor at the indicated concentrations for 30 min prior to treatment with indicated concentrations of As^III^ in combination with gamabufotalin, in the presence or absence of respective inhibitor for an additional 48 h.

### Cell Viability Assay

Following treatment for 48 h with various indicated concentrations of As^III^ and gamabufotalin, alone or in combination, cell viability was measured by the XTT assay as described previously (36, 38). Relative cell viability was expressed as the ratio of the absorbance at 450 nm of each treatment group against those of the corresponding untreated control group. Data are shown as mean ± standard deviation (SD) from more than three independent experiments. The IC_50_ value of the drug was calculated using GraphPad Prism ***^®^*** 6 software. In order to evaluate whether the two drugs generated synergistic, antagonistic, or additive effects, a combination index (CI) was determined as reported previously, using the computer software ComboSyn (Combosyn Inc. NJ, USA) for drug combinations and for general dose–effect analysis, which was developed by Chou (39, 40). The effect of the combination treatment was defined as a synergistic effect if CI < 1, an additive effect if CI = 1 or an antagonistic effect if CI > 1 (27, 41).

### Cell Cycle Analysis

After treatment with various indicated concentrations of As^III^ and gamabufotalin, alone or in combination, for 48 h, cell cycle analysis was performed using a FACSCanto™ flow cytometer (Becton Dickinson, CA, USA) according to the methods reported previously (19, 27, 37). Briefly, cells were washed twice with cold PBS, fixed with 1% paraformaldehyde/PBS on ice for 30 min, washed twice again with cold PBS, permeabilized in 70% (v/v) cold ethanol, and kept at −20°C for at least 4 h. Cell pellets were then washed twice with cold PBS after centrifugation (430 × g for 5 min at 4°C) and incubated with 0.25% Triton-X 100 for 5 min on ice. After centrifugation (430 × g for 5 min at 4°C) and washing with PBS, cells were resuspended in 500 µl of PI/RNase A/PBS (5 µg/ml of PI and 0.1% RNase A in PBS) and incubated for 30 min in the dark at room temperature. A total of 10,000 events were acquired, and FACSDiva™ software (v6.0; BD Biosciences) and ModFit LT™ v3.0 (Verity Software House, Inc., Topsham, ME, USA) were used to calculate the number of cells at G_2_/M phase fraction.

### Western Blot Analysis

For preparation of the protein samples, cell pellets (1–2 × 10^6^ cells per 110 μl buffer) were suspended in Laemmli buffer containing 100 mM DTT, 2 μg/ml leupeptin, 2 μg/ml aprotinin, 1 μg/ml pepstatin, and 1 mM PMSF. Cell suspensions were sonicated (Qsonica, LLC, Newtown, CT, USA) with 10 short bursts of 2 s followed by intervals of 2 s for cooling. The suspensions were then kept in an ice bath. Sonicated cells were heated in 95°C for 5 min, and then centrifuged at 13,000 × g for 15 min at 4°C. Protein concentrations of the supernatant were determined by Bradford’s method using the protein assay dye reagent (Bio-Rad Laboratories, Inc.) according to the manufacturer’s instructions, using BSA as standard. Western blot analysis was carried out according to a method previously described (37, 41). Briefly, protein samples (10–20 μg protein/lane) were separated on a sodium dodecyl sulfate polyacrylamide gel electrophoresis, followed by transferring to a polyvinylidene difluoride (PVDF) membrane, which was then blocked with 5% skim milk/PBST (PBS containing 0.5% Tween-20) for 1 h at room temperature. Protein bands were detected using the following primary antibodies: Mouse anti-human β-actin (1:5,000 dilution; cat. no. A-5441; Sigma-Aldrich; Merck KGaA, Darmstadt, Germany), rabbit anti-human cdc25C (1:1,000 dilution; cat. no. 4688), mouse anti-human Cyclin B1 (1:2,000 dilution; cat. no. 4135), mouse anti-human cdc2 (1:1,000 dilution; cat. no. 9116), mouse anti-human survivin (1:1000 dilution; cat. no. 2802), rabbit anti-human phospho-p38 (Thr180/Tyr182, 1:1,000 dilution; cat. no. 9211), and p38 (1:1,000 dilution; cat. no. 9212; all from Cell Signaling Technology, Inc., Danvers, MA, USA). PVDF membranes containing blotted protein bands were incubated overnight with the respective primary antibody at 4°C, followed by incubation with an appropriate horseradish peroxidase-conjugated secondary antibody (anti-mouse IgG, 1:3,000 dilution, cat. no. A5906; anti-rabbit IgG, 1:3,000 dilution, cat. no. A0545; both from Sigma-Aldrich; Merck KGaA) for 1 h at room temperature, and then detected with an enhanced chemiluminescence (ECL) analysis system (Amersham Pharmacia Biotech, Buckinghamshire, UK). Relative amounts of the immunoreactive proteins were calculated from the density of the gray level on a digitized image using a program, NIH Image 1.60.

### Lactate Dehydrogenase (LDH) Assay

Following treatment for 48 h with indicated concentrations of As^III^ and gamabufotalin, alone or in combination, LDH leakage from U-87 cells was measured using a LDH cytotoxicity detection kit (Wako Pure Chemical Industry) according to the method previously described with slight modifications (19, 36). Briefly, culture medium served as the negative control (NC). Culture supernatants (S) were collected by centrifugation at 450 × g for 5 min at 4°C and stored at −80°C until use. Cultured cells without treatment were lysed in the culture medium containing 0.2% Tween 20, and mixed aggressively using a vortex mixer, followed by the centrifugation at 12,000 × g for 10 min at 4°C and the cell lysate was used as the positive control (PC). In order to avoid an influence of Tween 20, culture medium containing 0.2% Tween 20 served as the negative control for PC and was referred to as NCT. Samples were diluted 16-fold with PBS and 50 μl of the diluted solution was transferred into wells of a 96-well plate. LDH activities were determined by adding 50 μl of “reaction reagent” from the kit, followed by incubation at room temperature for 30 min. The reaction was stopped by the addition of 100 μl of “stopping solution” provided with the kit at room temperature, and the absorbance at 560 nm was measured with a microplate reader (EMax*^®^* Plus, Molecular Devices, CA, USA). Cell damage was calculated as a percentage of LDH leakage from damaged cells using the following formula: LDH leakage (%) = (S-NC)/(PC-NCT) × 100. Data are shown as means and SD from three independent experiments.

### Statistical Analysis

Experiments were independently repeated three times, and the results were shown as the means ± standard deviation (SD) of three assays. Statistical analysis was conducted using one-way ANOVA followed by Dunnett’s post hoc test. A probability level of p < 0.05 was considered to indicate a statistically significant difference.

## Results

### Cytotoxic Effects of As^III^ and Gamabufotalin Against Human Glioblastoma Cell Lines U-87 and U-251

**Figure 1:**
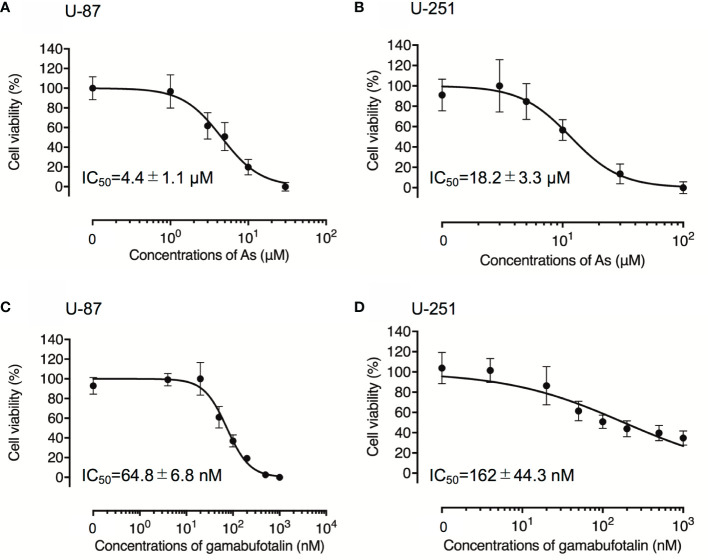
Respective cytotoxic effect of As^III^ and gamabufotalin against human glioblastoma cell lines U-87 and U-251. Cell viability was determined by XTT assay after the treatment with various concentrations of As^III^ alone [(1, 3, 5, 10, and 30 µM) **(A)**; (3, 5, 10, 30 and 100 µM) **(B)**], gamabufotalin alone [4, 20, 50, 100, 200, 500, and 1,000 nM **(C, D)**] for 48 h. Relative cell viability was calculated as the ratio of the absorbance at 450 nm of each treatment group against those of the corresponding untreated control group. Data are shown as the means ± SD (n ≥ 3). As, As^III^.

Although treatment with various concentrations of As^III^ for 48 h exhibited cytotoxic activities against U-87 and U-251 cells in a similar dose-dependent manner, the IC_50_ value of As^III^ in U-87 was approximately only one-fourth of that in U-251 cells (4.4 ± 1.1 µM in U-87; 18.2 ± 3.3 µM in U-251; p < 0.001) (**Figures 1A, B**). Treatment with gamabufotalin for 48 h also exhibited a dose-dependent cytotoxicity against U-87 cells with IC_50_ value of 64.8 ± 6.8 nM (**Figure 1C**). Interestingly, an untypical sigmoid growth inhibition model was observed in U-251 cells treated with gamabufotalin for 48 h, since the growth inhibition rates of the drug at concentrations >100 nM were very close to each other (**Figure 1D**). The IC_50_ value of gamabufotalin in U-87 cells was 2.5 times less than that in U-251 cells (64.8 ± 6.8 nM in U-87; 162 ± 44.3 nM in U-251; p < 0.05) (**Figures 1C, D**). These results indicated that U-87 cells were more sensitive to the cytotoxicity of both As^III^ and gamabufotalin, compared to U-251 cells.

### G_2_/M Arrest-Inducing Activity of As^III^ and Gamabufotalin in U-87 and U-251 Cells

**Figure 2:**
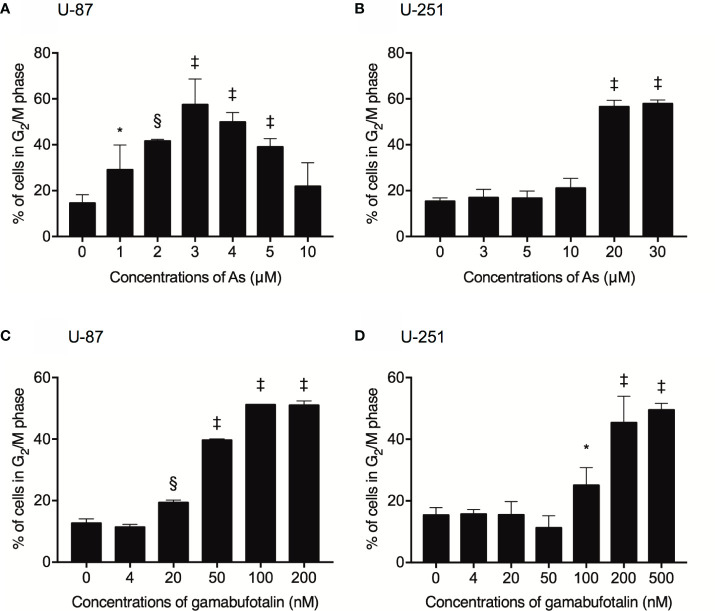
Respective G_2_/M arrest-inducing activity of As^III^ and gamabufotalin in U-87 and U-251 ells. Following treatment for 48 h with various concentrations of As^III^ (1, 2, 3, 4, 5, 10, 20, and 30 µM) alone **(A, B)**, gamabufotalin (4, 20, 50, 100, 200, and 500 nM) alone **(C, D)**, cell cycle profiling was performed by FACSCanto flow cytometer as described under *Materials and Methods*. A total of 10,000 events were acquired, and FACSDiva™ software and ModFit LT™ v3.0 were used to calculate the number of cells at G_2_/M phase fraction. Results are shown as the means ± SD (n ≥ 3). Significant difference between control and treatment groups are shown (*p < 0.05; ^§^p < 0.001; ^‡^p < 0.0001 *vs.* control). As, As^III^.

Treatment with various concentrations of As^III^ for 48 h exhibited a dose-dependent biphasic effect on G_2_/M arrest of U-87 cells, in which G_2_/M arrest-inducing activity of As^III^ was first observed at the concentration starting from 1 μM As^III^, and reached the maximum at the concentrations of 3 μM As^III^, then declined with increasing concentrations of As^III^ (**Figure 2A** and **Supplementary Figure 1**). In comparison, only relatively high concentrations of As^III^ (20 and 30 μM) prominently induced G_2_/M arrest in U-251 cells (**Figure 2B** and **Supplementary Figure 2**). Notably, a sub-G_1_ peak was concomitantly detected following treatment with relatively high concentrations of As^III^ (20 and 30 μM) in U251 cells (**Supplementary Figures 2E, F**). Exposure to gamabufotalin for 48 h also caused G_2_/M arrest of U-87 cells in a dose-dependent manner, and its G_2_/M arrest-inducing activity reached a plateau at the concentrations of 100 nM (**Figure 2C** and **Supplementary Figure 3**). In comparison, the G_2_/M arrest-inducing activity of gamabufotalin in U-251 cells was observed at the concentrations starting from 100 nM, and reached a plateau at the concentrations of 200 nM (**Figure 2D** and **Supplementary Figure 4**).

### Synergistic Cytotoxic Effect of As^III^ and Gamabufotalin in Glioblastoma Cell Line U-87 but Not U-251

**Figure 3:**
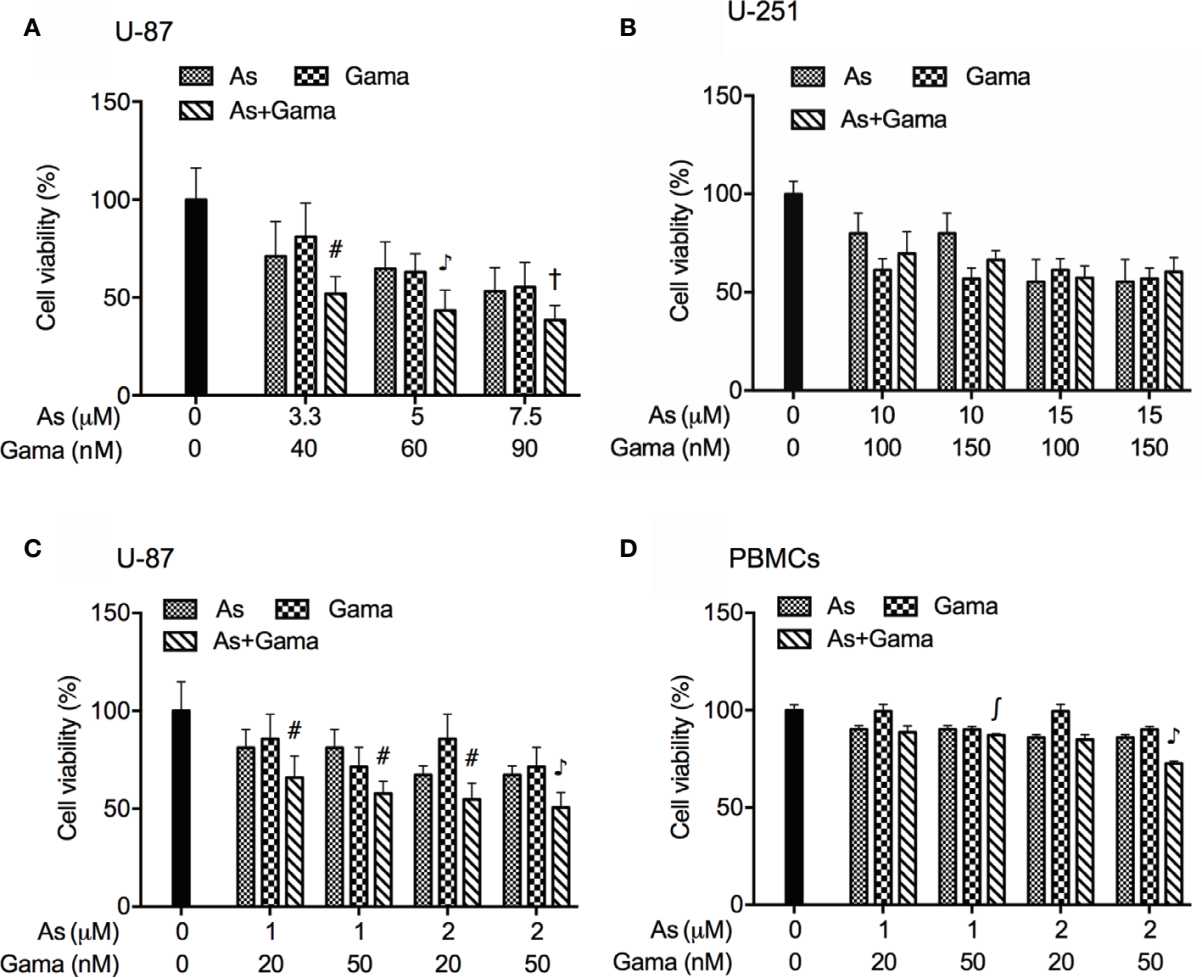
Synergistic cytotoxic effect of As^III^ and gamabufotalin in U-87 but not in U-251 cells. **(A)** U-87 cells were treated with relatively high concentrations of As^III^ (3.3, 5, and 7.5 µM) and gamabufotalin (40, 60, and 90 nM), alone or in combination. **(B)** U-251 cells were treated with relatively high concentrations of As^III^ (10 and 15 µM) and gamabufotalin (100 and 150 nM), alone or in combination. U-87 **(C)** and PBMCs **(D)** were treated with relatively low concentrations of As^III^ (1 and 2 µM) and gamabufotalin (20 and 50 nM), alone or in combination. Following the aforementioned treatment for 48 h, cell viability was determined by XTT assay. Relative cell viability was calculated as the ratio of the absorbance at 450 nm of each treatment group against those of the corresponding untreated control group. Data are shown as the means ± SD (n ≥ 3). ^∫^p < 0.05; ^#^p < 0.01; ^†^p < 0.001; ^♪^p < 0.0001 *vs.* each alone. As, As^III^; Gama, gamabufotalin.

In order to evaluate if the two drugs generated synergistic, antagonistic, or additive effects, based upon the IC_50_ values of each drug, the two-drug combination was determined according to the median-effect method of Chou (39, 40). As shown in **Figure 3A**, the combined treatment was significantly more cytotoxic than either drug alone in U-87 cells, and the values of combination index (CI) were <1 (**Table 1**), indicating the two drugs worked in a synergistic manner. However, similar combination effect was not observed in U-251 cells (**Figure 3B**), reconfirming that U-87 was highly susceptible to the combined regimen in comparison to U-251.

**Table 1:** CI values of the combination of relatively high concentrations of As^III^ and gamabufotalin in U-87 cells.

As^III^ (μM)	Gamabufotalin (nM)	Fa	CI value
3.3	40	0.52	0.77270
5	60	0.61	0.80496
7.5	90	0.69	0.86192

CI < 1 represents synergism. As, As^III^; Fa, the effect levels; CI, combination index.

Considering that G_2_/M arrest was significantly induced by 1 and 2 μM As^III^ (**Figure 2A**), both of which were considered as clinically achieved concentrations of As^III^, and by 20 and 50 nM gamabufotalin (**Figure 2C**), respectively, the cytotoxicity of relatively low concentrations of As^III^ (1 and 2 μM) and gamabufotalin (20 and 50 nM), alone or in combination, were further explored in U-87 cells in detail. As shown in **Figure 3C**, a modest but statistically significant decrease in cell viability was observed in the cells following treatment for 48 h with each single drug, and was further significantly intensified by their combination. The values of combination index (CI) of all combined treatment group were <1 (**Table 2**), indicating that the two drugs worked in a synergistic manner even at their relatively low concentrations. In comparison, under the same treatment conditions, only a modest growth inhibition (less than 15%) was induced by each single drug in PBMCs (**Figure 3D**). Furthermore, as compared to the growth inhibition rates ranging from 45 to 50% in U-87 cells, much lower inhibition rates ranging from 15 to 27% were observed in PBMCs (**Figures 3C, D**), indicating selective cytocidal effect of the combined regimen.

**Table 2:** CI values of the combination of relatively low concentrations of As^III^ and gamabufotalin in U-87 cells.

As (μM)	Gamabufotalin (nM)	Fa	CI value
1	20	0.34	0.77496
1	50	0.42	0.87181
2	20	0.45	0.79036
2	50	0.49	0.90709

CI < 1 represents synergism. As, As^III^; Fa, the effect levels; CI, combination index.

### Effects of Relatively Low Concentrations of As^III^ in Combination With Gamabufotalin on G_2_/M Arrest and the Expression Level of Cell Cycle Related-Proteins in U-87 Cells

**Figure 4:**
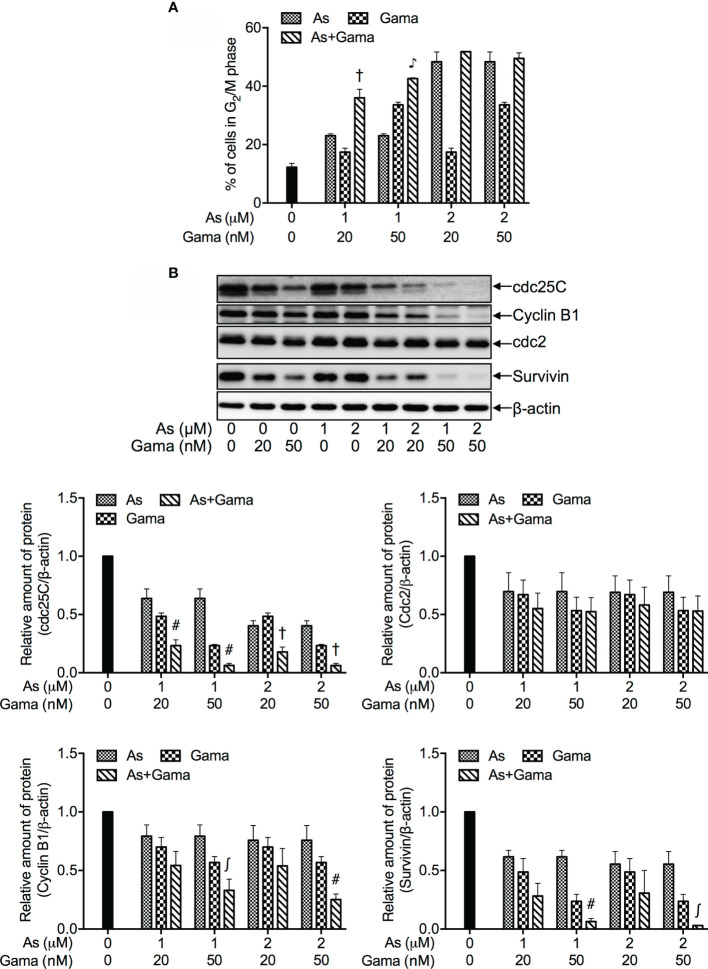
Effects of relatively low concentrations of As^III^ in combination with gamabufotalin on G_2_/M arrest and the expression level of cell cycle related-proteins in U-87 cells. Following treatment for 48 h with As^III^ (1 and 2 µM) and gamabufotalin (20 and 50 nM), alone or in combination, cell cycle profiling was performed by FACSCanto flow cytometer as described under *Materials and Methods*. A total of 10,000 events were acquired, and FACSDiva™ software and ModFit LT™ v3.0 were used to calculate the number of cells at G_2_/M phase fraction **(A)**. Results are shown as the means ± SD (n ≥ 3). After the same treatment, the expression profiles of cell cycle-associated proteins were analyzed using western blotting. A representation image of the expression profile of each protein is shown from three independent experiments **(B)**. The expression levels were expressed as the ratios between each targeted protein and β-actin protein expression levels, and were compared with those of control group. ^∫^p < 0.05; ^#^p < 0.01; ^†^p < 0.001; ^♪^p < 0.0001 *vs.* each alone. As, As^III^; Gama, gamabufotalin.

Consistent with results in **Figures 2A, C**, a dose-dependent G_2_/M arrest was induced by each single drug, and was further significantly augmented by 1 μM As^III^ in combination with 20 and 50 nM gamabufotalin, respectively (**Figure 4A** and **Supplementary Figure 5**). Probably due to the high potency of G_2_/M arrest-inducing activity of 2 μM As^III^, only a slight enhancement in its activity was observed by the addition of 20 or 50 nM gamabufotalin (**Figure 4A**).

As shown in **Figure 4B**, in comparison to control group, a clear downregulation of the expression of cdc25C was induced by each single drug, and was further significantly intensified by their combination following treatment for 48 h with As^III^ (1, 2 μM) and gamabufotalin (20, 50 nM), alone or in combination. A modest but significant downregulation of the expression level of Cyclin B1 was induced by 2 μM As^III^, and 20, 50 nM gamabufotalin, respectively. A slight decrease of the expression level of Cyclin B1 induced by 1 μM As^III^ was significantly intensified by the addition of 50 nM gamabufotalin. Similar phenomena were also observed in the cells treated with 2 μM As^III^ combined with 50 nM gamabufotalin. Moreover, a trend towards a downregulation of Cyclin B1 was observed in the treatment of 1 or 2 μM As^III^ combined with 20 nM gamabufotalin, although there was no significant difference between each single drug and their combination. In addition, the clinically achieved concentrations of As^III^ (1 and 2 µM) also modestly and significantly suppressed the expression level of survivin. In comparison, the addition of either 20 nM or 50 nM gamabufotalin appeared to strongly downregulate the expression of survivin. Of note, as compared to 20 nM gamabufotalin, the addition of 50 nM gamabufotalin more efficiently intensified As^III^-mediated survivin downregulation. Moreover, each single drug (except for 1 μM As^III^) modestly and significantly suppressed the expression of cdc2, and their combination slightly strengthened the suppression.

### Enhanced LDH Release in U-87 Cells Treated With Relatively Low Concentrations of As^III^ Combined With Gamabufotalin

**Figure 5:**
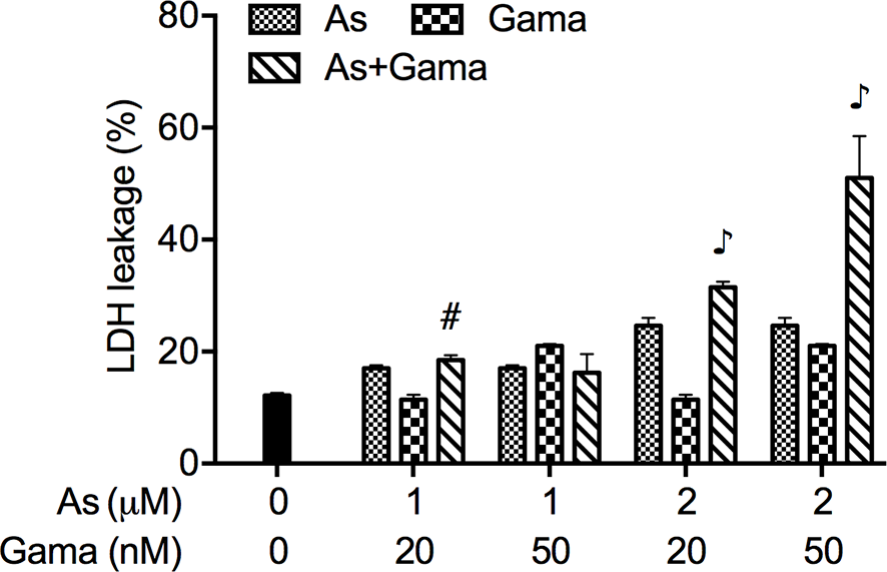
Enhanced LDH release in U-87 cells treated with relatively low concentrations of As^III^ combined with gamabufotalin. Following treatment for 48 h with As^III^ (1 and 2 µM) and gamabufotalin (20 and 50 nM), alone or in combination, LDH leakage was measured using the LDH-Cytotoxic test kit as described under Materials and methods. Results are shown as the means ± SD (n ≥ 3). ^#^p < 0.01; ^♪^p < 0.0001 *vs.* each alone. As, As^III^; Gama, gamabufotalin.

The release of LDH provides an accurate measure of the cell membrane integrity and cell viability (27, 36). Following treatment with relatively low concentrations of As^III^ (1 and 2 μM) and gamabufotalin (20 and 50 nM), alone or in combination, for 48 h, LDH leakage analysis was conducted to examine whether the combined treatment directly affected cell membrane integrity. As shown in **Figure 5**, a measurable level of LDH leakage was induced by each single drug except for 20 nM gamabufotalin. The exposure of 2 μM As^III^ in combination with either 20 or 50 nM gamabufotalin further prominently enhanced the LDH leakage. Additionally, a slight but significant increase in the LDH leakage was induced by 1 μM As^III^ combined with 20 nM gamabufotalin in comparison with each single drug. Intriguingly, similar phenomenon was not observed in the treatment of 1 μM As^III^ in combination with 50 nM gamabufotalin, which is might be a result of relatively high efficiency of 50 nM gamabufotalin itself to induce LDH leakage.

### Prosurvival Role of p38 MAPK in the Cytotoxicity of U-87 Cells Treated With the Combination of As^III^ and Gamabufotalin

**Figure 6:**
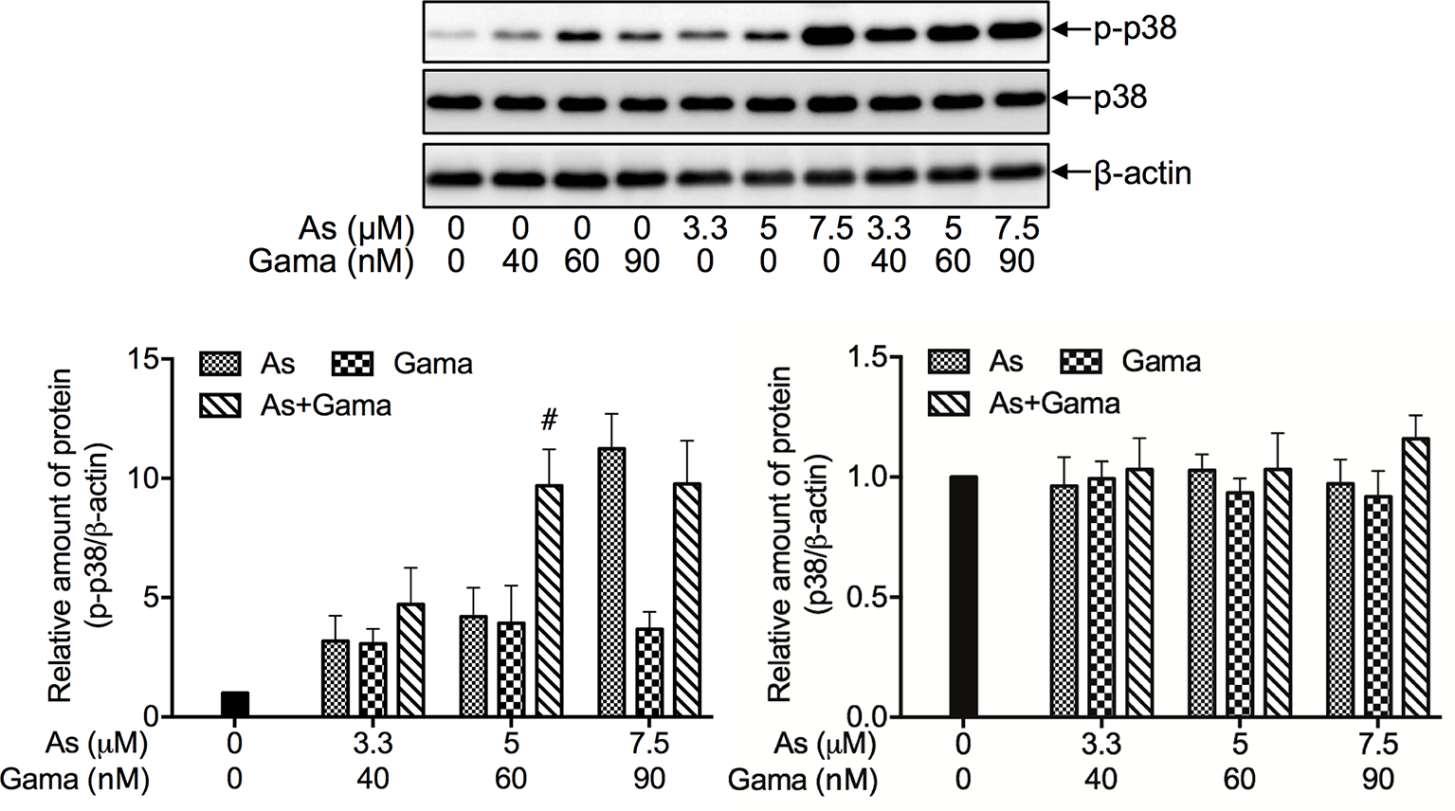
Effect of relatively high concentrations of As^III^ and gamabufotalin on the expression level of phospho-p38 and its total form in U-87 cells. Following treatment for 48 h with various concentrations of As^III^ (3.3, 5, 7.5 µM) and gamabufotalin (40, 60, 90 nM), alone or in combination, the expression profiles of phospho-p38 (p-p38) and p38 were analyzed using western blotting. A representation image of the expression profile of each protein is shown from three independent experiments. The expression levels were expressed as the ratios between each targeted protein and β-actin protein expression levels, and were compared with those of control group. A p value less than 0.05 was considered as statistically significant (^#^p < 0.01 *vs.* each alone). As, As^III^; Gama, gamabufotalin.

**Figure 7:**
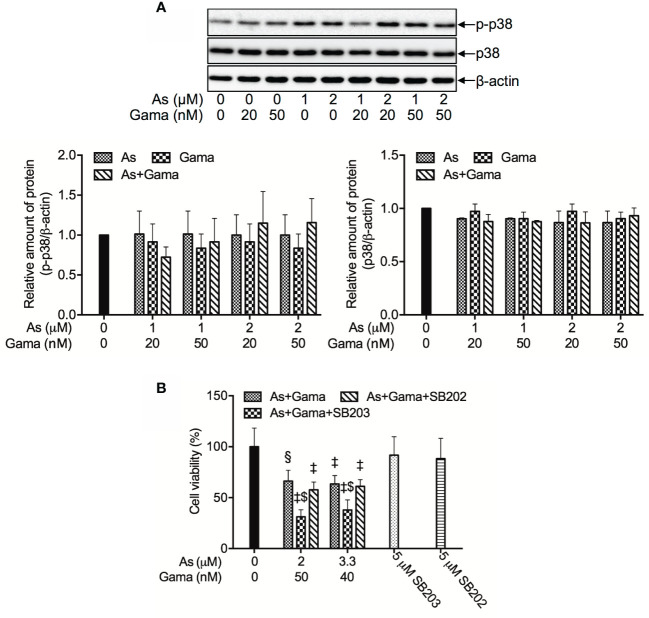
Prosurvival role of p38 MAPK in the cytotoxicity of U-87 cells treated with the combination of As^III^ and gamabufotalin. **(A)** Following treatment for 48 h with relatively low concentrations of As^III^ (1, 2 μM) and gamabufotalin (20, 50 nM), alone or in combination, the expression profiles of phospho-p38 (p-p38) and p38 were analyzed using western blotting. A representation image of the expression profile of each protein is shown from three independent experiments. The expression levels were expressed as the ratios between each targeted protein and β-actin protein expression levels, and were compared with those of control group. **(B)** Following treatment for 48 h with the combined regimen of 2 μM As^III^ + 50 nM gamabufotalin; 3.3 μM As^III^ + 40 nM gamabufotalin, in the presence of absence of 5 μM SB203580, a specific inhibitor for p38 MAPK and its negative control SB202474, cell viability was determined by XTT assay. Relative cell viability was calculated as the ratio of the absorbance at 450 nm of each treatment group against those of the corresponding untreated control group. Data are shown as the means ± SD (n ≥ 3). A p value less than 0.05 was considered as statistically significant (^§^p < 0.001; ^‡^p < 0.0001 vs. control. ^$^p < 0.001 vs. As+Gama and As+Gama+SB202474). As, As^III^; Gama, gamabufotalin; SB203, SB203580; SB202, SB202474. The images of beta-actin are identical to that in **Figure 4B** since the same experiment samples were used to analyze.

Since we have recently demonstrated a prosurvival role for p38 MAPK in glioblastoma cells treated with active bufadienolide compounds including gamabufotalin (19, 36), the activation of p38 MAPK and its possible role associated with cell viability were explored in U-87 cells following the treatment with the combination of either relatively high or low concentrations of As^III^ and gamabufotalin. As shown in **Figure 6**, the expression of phospho-p38 (p-p38) was modestly and clearly upregulated by gamabufotalin and As^III^ (3.3, 5 μM), respectively, and further strengthened by the combination of 3.3 μM As^III^ + 40 nM gamabufotalin, and 5 μM As^III^ + 60 nM gamabufotalin. A dramatic upregulation of the expression level of p-p38 was induced by 7.5 μM As^III^, and the further enhancement was not observed by the addition of 90 nM gamabufotalin. Moreover, no alteration of the expression level of p38 was observed regardless of treatment with each single drug alone or their combination. As shown in **Figure 7A**, in comparison to a significant upregulation of p-p38 expression triggered by relatively high concentrations of As^III^ and gamabufotalin, almost no alteration of p-p38 expression was observed in the cells following the treatment with 1 μM As^III^ combined with 20 and 50 nM gamabufotalin, respectively. In addition, only a slight increase in the expression level of p-p38 was observed in the cells following the treatment with 2 μM As^III^ combined with 20 or 50 nM gamabufotalin in comparison with each single drug. Again, almost no alteration of the expression level of p38 was observed regardless of treatment with each single drug alone or their combination.

In order to clarify whether p38 MAPK is directly involved in the cytotoxicity of the combined regimen, alterations of the cell viability were determined in U-87 cells following treatment for 48 h with the combination of As^III^ and gamabufotalin (2 μM As^III^ + 50 nM gamabufotalin; 3.3 μM As^III^ + 40 nM gamabufotalin) in the presence or absence of 5 μM SB203580, a specific inhibitor of p38 MAPK. Consistent with the results in **Figure 3**, exposure to the aforementioned combined regimen significantly reduced cell viability by approximately 50% (**Figure 7B**). The efficacy of each combined regimen was significantly intensified by the addition of SB203580, whereas the similar phenomena were not observed by the addition of SB202474, a negative control of SB203580 (**Figure 7B**), indicating the critical role of p38 MAPK for cell survival. Moreover, neither SB203580 nor SB202474 itself affected the cell viability.

### Involvement of Autophagic Cell Death in the Cytotoxicity of U-87 Cells Treated With the Combination of As^III^ and Gamabufotalin

**Figure 8:**
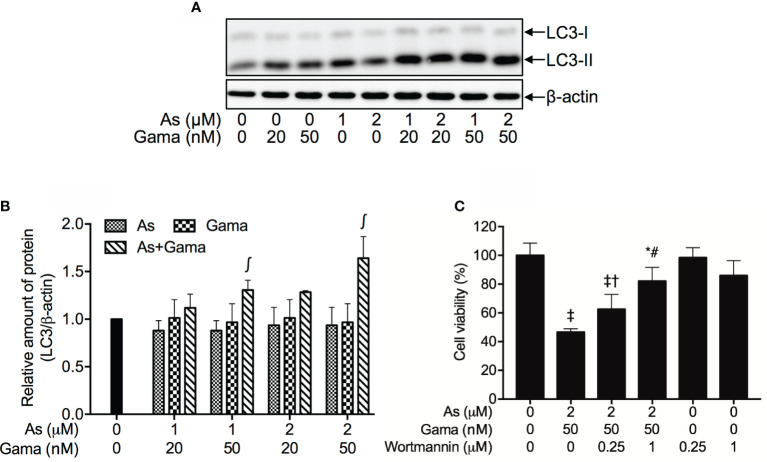
Involvement of autophagic cell death in the cytotoxicity of U-87 cells treated with the combination of As^III^ and gamabufotalin. Following treatment for 48 h with As^III^ (1, 2 μM) and gamabufotalin (20, 50 nM), alone or in combination, the expression profiles of LC3 were analyzed using western blotting. A representation image of the expression profile of LC3 is shown from three independent experiments **(A)**. The expression levels were expressed as the ratio between LC3 protein and β-actin protein expression levels, and were compared with those of control group **(B)**. Cell viability was determined by XTT assay after treatment for 48 h with the combination of 2 μM As^III^ and 50 nM gamabufotalin in the presence or absence of wortmannin (0.25, 1 μM) **(C)**. Data are shown as the means ± SD (n ≥ 3). A p value less than 0.05 was considered as statistically significant (^∫^p < 0.05 vs. each alone; ^*^p < 0.05; ^‡^p < 0.0001 vs. control; ^†^p < 0.05; ^#^p < 0.0001 vs. As+Gama). As, As^III^; Gama, gamabufotalin. The images of beta-actin are identical to that in **Figure 4B** since the same experiment samples were used to analyze.

As shown in **Figures 8A, B**, compared to control group and each single drug, a modest upregulation of the expression level of LC3, an autophagic marker, was clearly induced by the treatment of 1 and 2 µM As^III^ when combined with 20 nM gamabufotalin, respectively. Furthermore, both 1 µM As^III^+50 nM gamabufotalin and 2 µM As^III^+50 nM gamabufotalin significantly upregulated the expression level of LC-3 in comparison to each single drug treatment, and the combination of 2 µM As^III^+50 nM gamabufotalin showed greater potency.

Since induction of autophagy by various anticancer drugs has been suggested to be a potential therapeutic strategy for cancer including glioblastoma (42–46), wortmannin, a potent autophagy inhibitor, was employed to further confirm whether the induction of autophagy contributed to the combined treatment-induced cell death. As shown in **Figure 8C**, following treatment with 2 μM As^III^ plus 50 nM gamabufotalin for 48 h, a significant decrease in cell viability was confirmed again in U-87 cells. The addition of wortmannin, however, significantly rescued the cell from toxicity caused by the combined regimen in a dose-dependent manner. Moreover, the cell viability was almost not altered by wortmannin alone.

## Discussion

Results from this study clearly demonstrated that As^III^ and gamabufotalin exhibited cytotoxicity against glioblastoma cell lines U-87 and U-251, and that U-87 cells were more sensitive to the cytotoxicity of both drugs in comparison to U-251 cells (**Figure 1**). Previous studies have demonstrated that U-87 cells express wild type p53, known as a tumor suppressor, whereas U-251 cells harbor p53 mutant (12, 44, 47). An intact p53 protein function has been implicated in the cytotoxicity of As_2_O_3_ in glioblastoma cells as evidenced by the fact that U-87 cells were more susceptible to the drug than T98G, another glioblastoma cell line harboring p53 mutant (12). Collectively, the p53 status could be attributed to the differential susceptibility of U-87 and U-251 cells to either As^III^ or gamabufotalin, although further studies will be needed to clarify whether both drugs affect the expression and function of p53 in these cells.

Our cell cycle analysis results further demonstrated that a dose-dependent biphasic effect of As^III^ on G_2_/M arrest was interestingly observed in U-87 cells, in which G_2_/M arrest-inducing activity of As^III^ was observed at the concentration starting from 1 μM As^III^, and reached the maximum at the concentrations of 3 μM As^III^, then declined with increasing concentrations of As^III^ (**Figure 2A**). In comparison, a marked G_2_/M arrest along with sub-G_1_ peak, known as a hallmark of apoptosis, was concomitantly observed in U-251 cells following the exposure to relatively high concentrations of As^III^ (20 and 30 μM) (**Figure 2B** and **Supplementary Figure 2**), suggesting that besides G_2_/M arrest, apoptosis induction is also implicated in the cytotoxicity of As^III^ in the cells. In fact, previous reports have demonstrated that As_2_O_3_ inhibited the growth of glioblastoma cell lines including U-87 and U-251 *via* apoptosis induction as well as G_2_/M arrest (12–14). Considering the higher susceptibility of U-87 cells to As^III^, we hypothesized that the drop in the G_2_/M population caused by >3 μM As^III^ and the lack of sub-G_1_ peak might be due to a loss of a portion of damaged cells, which probably turned into dead cell debris and were too small to be detected, as a result of intensive cytotoxicity of As^III^. The hypothesis testing and further studies on whether apoptosis induction occurs and links to cell cycle arrest in both U-87 and U-251 cells treated with As^III^ and gamabufotalin, alone or in combination, are ongoing in our laboratory. In the current study, instead of focusing on the mechanisms underlying the biphasic effect of As^III^ on G_2_/M arrest in U-87 cells, a promising result showing that G_2_/M arrest was significantly induced by clinically achieved concentrations of As^III^ (1 and 2 μM) encouraged us to explore the effects of the relatively low concentrations of As^III^ in combination with gamabufotalin against U-87 cells as mentioned below.

Active bufadienolide compounds, including bufalin, arenobufagin and hellebrigenin, have been demonstrated to induce G_2_/M arrest in U-87 or U-251 (19, 24, 48). In line with these previous findings, treatment with gamabufotalin caused G_2_/M arrest in both two cells (**Figures 2C, D**, **Supplementary Figures 3** and **4**). Of note, the G_2_/M arrest-inducing activity of gamabufotalin was more potent in U-87 compared to that in U251 as evidence by the fact that G_2_/M arrest already reached a plateau at the concentrations of 100 nM gamabufotalin in U-87 cells, whereas a clear G_2_/M arrest just began to appear at the same concentration in U-251 cells (**Figures 2C, D**, **Supplementary Figures 3** and **4**). These results thus reconfirmed the higher susceptibility of U-87 cells to gamabufotalin, and further suggested that the G_2_/M arrest play a critical role in the cytotoxicity of both As^III^ and gamabufotalin in both cancer cells.

It is noteworthy that like the treatment with relatively high concentrations of As^III^ (3.3, 5, 7.5 μM) and gamabufotalin (40, 60, 90 nM) (**Figure 3A**, **Table 1**), synergistic cytotoxic effects of clinically achieved concentrations of As^III^ (1, 2 μM) and gamabufotalin (20, 50 nM) were also successfully observed in U-87 cells (**Figure 3C**, **Table 2**). Most importantly, the same regimen was much less cytotoxic to PBMCs (**Figure 3D**), suggesting the selective cytocidal effects of the combined regimen and its possible beneficial effect in patient with glioblastoma. Moreover, enhanced G_2_/M arrest was observed in U-87 cells treated with the combined regimen of relatively low concentrations of As^III^ (1, 2 μM) and gamabufotalin (20, 50 nM), accompanied by a downregulation of cdc25C and Cyclin B1/cdc2 as well as survivin (**Figure 4** and **Supplementary Figure 5**). Several lines of evidence have demonstrated that in glioblastoma cell lines such as U-87 and U-251, downregulation of the expression levels of Cyclin B1 and cdc2 has been implicated in the G_2_/M arrest induced by various anticancer agents, including As_2_O_3_ (12, 14), active bufadienolide compounds such as hellebrigenin (19) and geldanamycin, an inhibitor of the chaperone activity of heat shock protein 90 (Hsp90) (49). Survivin is highly expressed in most human cancer cells including primary human glioblastoma cells (19, 30), and its inhibition has been considered as a compelling strategy for cancer therapy (50). Moreover, the expression of survivin has been suggested to be a useful biomarker for predicting the prognosis in glioblastoma patients (51). Previous studies have demonstrated that survivin expression peaks in the G_2_/M phase and rapidly declines in the G_1_ phase, and that survivin can be phosphorylated by cdc2 (50). In fact, our previous results have reported that exposure to active bufadienolide compounds including gamabufotalin resulted in the downregulation of survivin expression along with G_2_/M arrest in U-87 cells (19, 36). Taken together, these results suggested that G_2_/M arrest associated with downregulation of cdc25C, Cyclin B1/cdc2 and survivin largely contributed to the cytotoxic effect of As^III^ and gamabufotalin even at their relatively low concentrations.

Of note, LDH leakage was also induced by relatively low concentrations of As^III^ and gamabufotalin, and was further augmented by their combination, especially 2 μM As^III^ combined with 20 and 50 nM gamabufotalin, respectively (**Figure 5**). Similarly, a previous report demonstrated that As^III^ induced necrosis through a regulated, Bcl-xL-sensitive mitochondrial pathway in an APL NB4 cell line (52). Considering only small enhancement in G_2_/M arrest was observed in the combination of 2 μM As^III^ plus 20 or 50 nM gamabufotalin in comparison to each single drug (**Figure 4A**), the synergistic cytocidal effect of the combined regimen could be attributed to both G_2_/M arrest and LDH leakage.

Intriguingly, previous studies have demonstrated a prosurvival role for p38 MAPK in glioblastoma cells, including U-87 cells (19, 36, 53). Furthermore, phosphorylation of p38 MAPK has been suggested to be a prognostic marker for patients with high-grade glioma, and vandetanib combined with a p38 MAPK inhibitor may be a useful combination chemotherapy for patients with glioma (35). In line with these previous findings, activation of p38 MAPK pathway was observed in U-87 cells following treatment with As^III^ and gamabufotalin, alone or in combination, as evidenced by a clear increase in the expression level of phopho-p38, although the magnitude of increase was dependent on drug concentrations (**Figures 6** and **7**). Our results further demonstrated that the addition of a specific inhibitor of p38 MAPK enhanced the cytotoxicity of the combination of As^III^ and gamabufotalin, reconfirming its prosurvival role in the cells (**Figure 7B**). Taking the previous results and our observations into account, we thus hypothesized that combining a p38 MAPK inhibitor with the combined regimen of As^III^ and gamabufotalin may further improve the efficacy of these drugs, and may provide more therapeutic benefits to patients with glioblastoma, although the precise contribution of the p38 MAPK pathway warrants further investigation *in vitro* and *in vivo*. In addition, only modest induction of p38 MAPK activation by the clinically achieved concentrations of As^III^ in combination with gamabufotalin suggest that the combined regimen should be beneficial to avoid activating the prosurvival pathway in glioblastoma cells.

Induction of autophagic cell death by various chemotherapeutic agents has also considered as a potential therapeutic strategy for cancer (13, 43–45). In the current study, the autophagic cell death was confirmed by the significant upregulation of LC-3, an autophagic marker, in U-87 cells treated with the combined regimen of As^III^ and gamabufotalin (**Figures 8A, B**). The cytotoxicity of combined regimen was further significantly abrogated by the addition of wortmannin, a potent autophagy inhibitor (27, 41), in a dose-dependent manner (**Figure 8C**). In line with these current findings, autophagic cell death has been implicated in the cytotoxicity of As_2_O_3_ against several types of glioblastoma cells (13, 43, 45). Several research groups including us have also demonstrated that a number of bufadienolides such as bufalin, arenobufagin and gamabufotalin induce autophagy in numerous cancer cells including glioblastoma cells (36, 54, 55). Collectively, our results suggested for the first time that besides G_2_/M arrest and necrosis, autophagic cell death also partially contributed to cytotoxicity of the combined regimen of As^III^ and gamabufotalin.

Interestingly, Yoshimura and colleagues have demonstrated that As_2_O_3_ sensitizes glioblastoma to a Myc inhibitor-mediated growth inhibition, based on a study using patient-derived glioblastoma cancer stem-like cells and its xenograft model (56). Although gamabufotalin has been identified as an inhibitor of c-Myc in myeloma cell lines (57), our previous study demonstrated that treatment with gamabufotalin almost had no effect on the expression of c-Myc in U-87 cells (36). Given that c-Myc may serve as one of the candidate targets of cancer therapy (58, 59), future studies on how As^III^ in combination with gamabufotalin affects the c-Myc expression profile in a mouse xenograft model of human glioblastoma are warranted.

## Conclusion

Our results demonstrated that U-87 cells were highly susceptible to both As^III^ and gamabufotalin, alone or in combination, in comparison to U-251 cells. We further demonstrated that clinically achieved concentrations of As^III^ combined with gamabufotalin exhibited synergistic cytotoxic effect against U-87 cells, whereas showed much less cytotoxic to PBMCs, suggesting that the combined regimen could provide possible beneficial effect in patient with glioblastoma with high potency, selectivity and tolerability. G_2_/M arrest, necrosis as well as autophagic cell death induction appeared to cooperatively contribute to the synergistic cytotoxicity of As^III^ combined with gamabufotalin. Given that p38 MAPK serves an essential role in promoting glioblastoma cell survival, developing a possible strategy composed of As^III^, gamabufotalin and a p38 MAPK inhibitor with the aim of preventing the activation of the p38 MAPK pathway may provide novel insight into approaches designed to treat glioblastoma. Furthermore, the capacity of gamabufotalin to cross the blood-brain barrier is also of great concern and must warrant further investigation.

## Data Availability Statement

The original contributions presented in the study are included in the article/**Supplementary Material**. Further inquiries can be directed to the corresponding author.

## Ethics Statement

The studies involving human participants were reviewed and approved by the Institutional Review Board committee of Tokyo University of Pharmacy and Life Science. The patients/participants provided their written informed consent to participate in this study.

## Author Contributions

BY conceived and designed the study, and drafted the manuscript. KX and RS performed the experiments. JL, HH, MO, and NT assisted interpretation of the results with BY. All authors contributed to the article and approved the submitted version.

## Funding

This work was partially supported by The Japan Society for the Promotion of Science (JSPS) KAKENHI Grant to BY (Grant Numbers: 20K07136).

## Conflict of Interest

The authors declare that the research was conducted in the absence of any commercial or financial relationships that could be construed as a potential conflict of interest. 
