# Corrigendum: Cytotoxic Effects of Arsenite in Combination With Gamabufotalin Against Human Glioblastoma Cell Lines

**DOI:** 10.3389/fonc.2021.778834

**Published:** 2021-10-08

**Authors:** Bo Yuan, Kang Xu, Ryota Shimada, JingZhe Li, Hideki Hayashi, Mari Okazaki, Norio Takagi

**Affiliations:** ^1^ Laboratory of Pharmacology, Faculty of Pharmaceutical Sciences, Josai University, Sakado, Japan; ^2^ Department of Applied Biochemistry, Tokyo University of Pharmacy & Life Sciences, Hachioji, Japan; ^3^ Beijing Key Laboratory of Research of Chinese Medicine on Prevention and Treatment for Major Diseases, Experimental Research Center, China Academy of Chinese Medical Sciences, Beijing, China

**Keywords:** arsenite, gamabufotalin, glioblastoma, cell cycle arrest, p38 MAPK, autophagy, lactate dehydrogenase, combination therapy

In the original article, there was a mistake in the legend for **Figures 7A and 8** as published. **Beta-actin bands in Fig. 4B, 7A and 8 are identical in each figure legend**. The correct legend appears below.

**FIGURE 7** Prosurvival role of p38 MAPK in the cytotoxicity of U-87 cells treated with the combination of As^III^ and gamabufotalin. (A) Following treatment for 48 h with relatively low concentrations of As^III^ (1, 2 μM) and gamabufotalin (20, 50 nM), alone or in combination, the expression profiles of phospho-p38 (p-p38) and p38 were analyzed using western blotting. A representation image of the expression profile of each protein is shown from three independent experiments. The expression levels were expressed as the ratios between each targeted protein and β-actin protein expression levels, and were compared with those of control group. (B) Following treatment for 48 h with the combined regimen of 2 μM As^III^ + 50 nM gamabufotalin; 3.3 μM As^III^ + 40 nM gamabufotalin, in the presence of absence of 5 μM SB203580, a specific inhibitor for p38 MAPK and its negative control SB202474, cell viability was determined by XTT assay. Relative cell viability was calculated as the ratio of the absorbance at 450 nm of each treatment group against those of the corresponding untreated control group. Data are shown as the means ± SD (n ≥ 3). A p value less than 0.05 was considered as statistically significant (^§^p < 0.001; ^‡^p < 0.0001 vs. control. ^$^p < 0.001 vs. As+Gama and As+Gama+SB202474). As, As^III^; Gama, gamabufotalin; SB203, SB203580; SB202, SB202474. The images of beta-actin are identical to that in Figure 4B since the same experiment samples were used to analyze.

**FIGURE 8** Involvement of autophagic cell death in the cytotoxicity of U-87 cells treated with the combination of As^III^ and gamabufotalin. Following treatment for 48 h with As^III^ (1, 2 μM) and gamabufotalin (20, 50 nM), alone or in combination, the expression profiles of LC3 were analyzed using western blotting. A representation image of the expression profile of LC3 is shown from three independent experiments (A). The expression levels were expressed as the ratio between LC3 protein and β-actin protein expression levels, and were compared with those of control group (B). Cell viability was determined by XTT assay after treatment for 48 h with the combination of 2 μM As^III^ and 50 nM gamabufotalin in the presence or absence of wortmannin (0.25, 1 μM) (C). Data are shown as the means ± SD (n ≥ 3). A p value less than 0.05 was considered as statistically significant (^∫^p < 0.05 vs. each alone; ^*^p < 0.05; ^‡^p < 0.0001 vs. control; ^†^p < 0.05; ^#^p < 0.0001 vs. As+Gama). As, As^III^; Gama, gamabufotalin. The images of beta-actin are identical to that in Figure 4B since the same experiment samples were used to analyze.

The authors apologize for this error and state that this does not change the scientific conclusions of the article in any way. The original article has been updated.

## Publisher’s Note

All claims expressed in this article are solely those of the authors and do not necessarily represent those of their affiliated organizations, or those of the publisher, the editors and the reviewers. Any product that may be evaluated in this article, or claim that may be made by its manufacturer, is not guaranteed or endorsed by the publisher.

