# Supplementary material for: Cytotoxic Effects of Arsenite in Combination With Gamabufotalin Against Human Glioblastoma Cell Lines
Source: Front Oncol. 2021 Mar 16;11:628914. doi: 10.3389/fonc.2021.628914 (PMC8009626; doi:10.3389/fonc.2021.628914)
Supplement: Supplementary file 1 [file DataSheet_1.docx]

Supplementary Material

# Supplementary Data

Supplementary Material should be uploaded separately on submission. Please include any supplementary data, figures and/or tables. All supplementary files are deposited to FigShare for permanent storage and receive a DOI.

Supplementary material is not typeset so please ensure that all information is clearly presented, the appropriate caption is included in the file and not in the manuscript, and that the style conforms to the rest of the article. To avoid discrepancies between the published article and the supplementary material, please do not add the title, author list, affiliations or correspondence in the supplementary files.

# Supplementary Figures and Tables

For more information on Supplementary Material and for details on the different file types accepted, please see [here](http://home.frontiersin.org/about/author-guidelines#SupplementaryMaterial). Figures, tables, and images will be published under a Creative Commons CC-BY licence and permission must be obtained for use of copyrighted material from other sources (including re-published/adapted/modified/partial figures and images from the internet). It is the responsibility of the authors to acquire the licenses, to follow any citation instructions requested by third-party rights holders, and cover any supplementary charges.

## Supplementary Figures


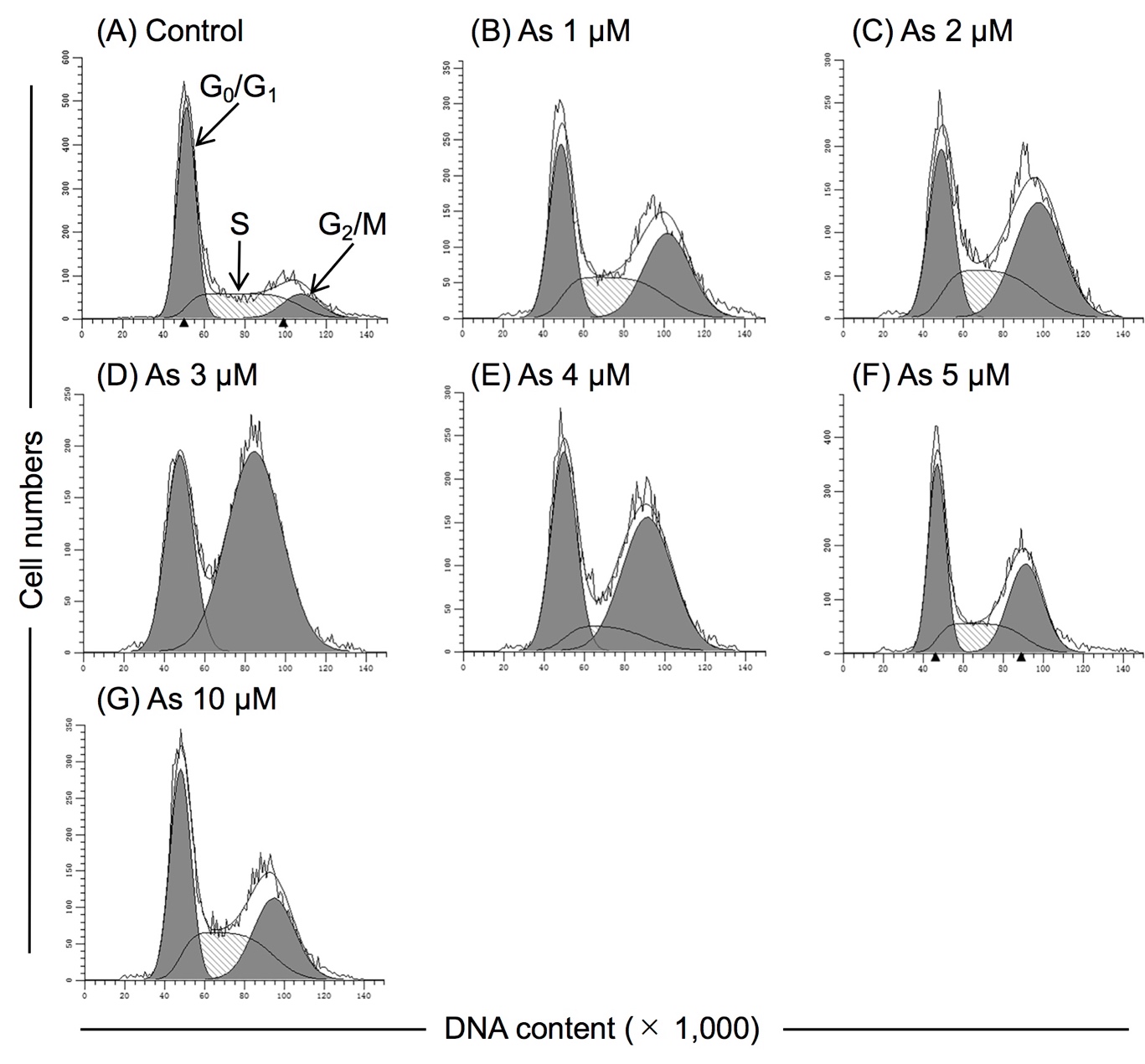


**Supplementary Figure 1.** Effects of As^III^ on the cell cycle profiling of U-87 cells. Following treatment with various concentrations of As^III^ (1, 2, 3, 4, 5 and 10 µM) for 48 h, cell cycle profiling was performed by FACSCanto flow cytometer as described under Materials and methods [(A)-(G)]. Analyzed data and profiles for each G_0_/G_1_ and G_2_/M phase using Diva software and ModFit LTTM ver. 3.0. are shown in the gray area. Cells at S phase are shown as shaded area. A representative FACS histogram from three separate experiments is shown. As, As^III^.


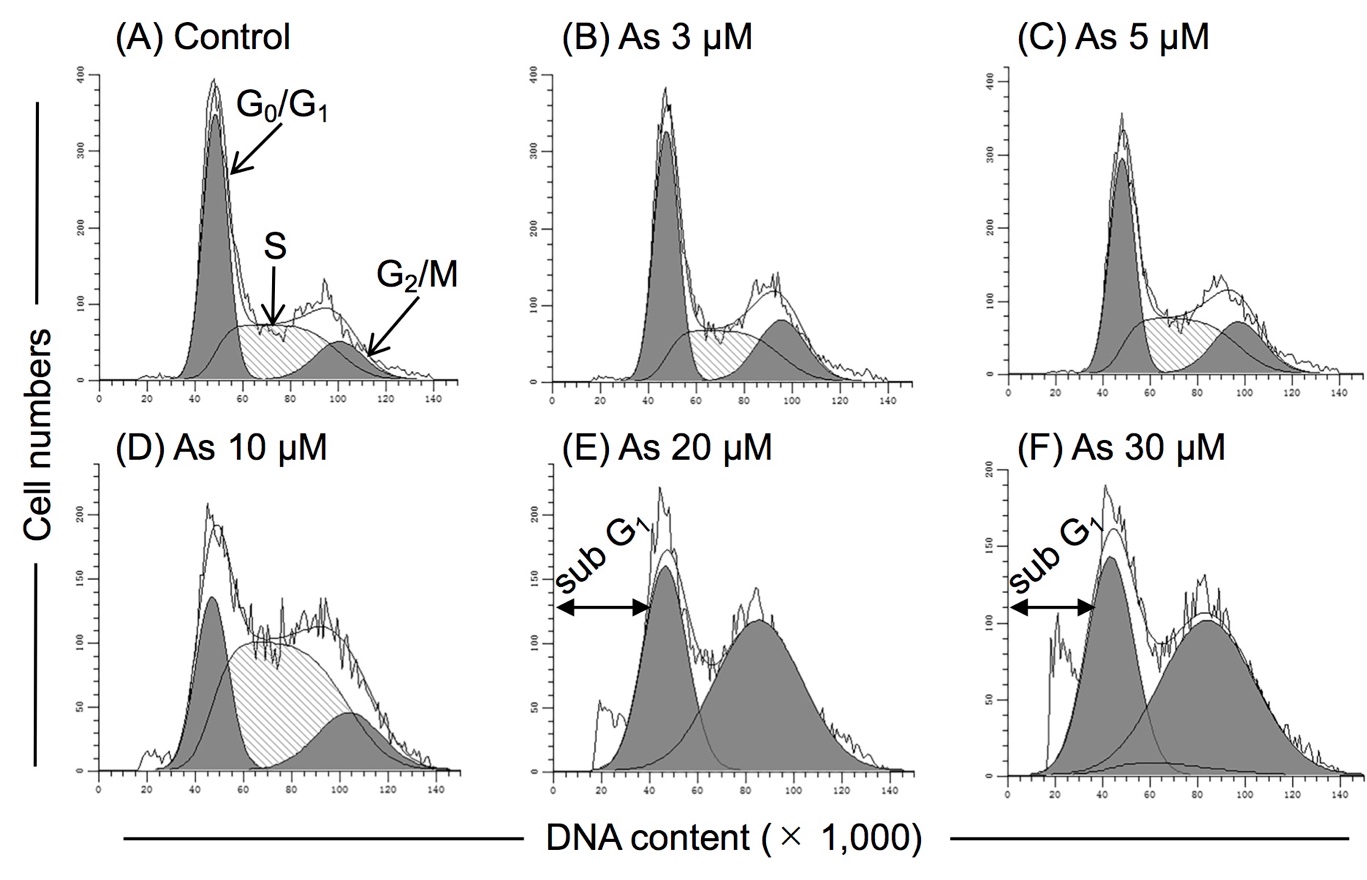


**Supplementary Figure 2.** Effects of As^III^ on the cell cycle profiling of U-251 cells**.** Following treatment with various concentrations of As^III^ (3, 5, 10, 20 and 30 µM) for 48 h, cell cycle profiling was performed by FACSCanto flow cytometer as described under Materials and methods [(A)-(F)]. Analyzed data and profiles for each sub-G_1_, G_0_/G_1_ and G_2_/M phase using Diva software and ModFit LT^TM^ ver. 3.0. are shown in the open (sub-G_1_ phase) and gray area (G_0_/G_1_ and G_2_/M phase). Cells at S phase are shown as shaded area. A representative FACS histogram from three separate experiments is shown. As, As^III^.


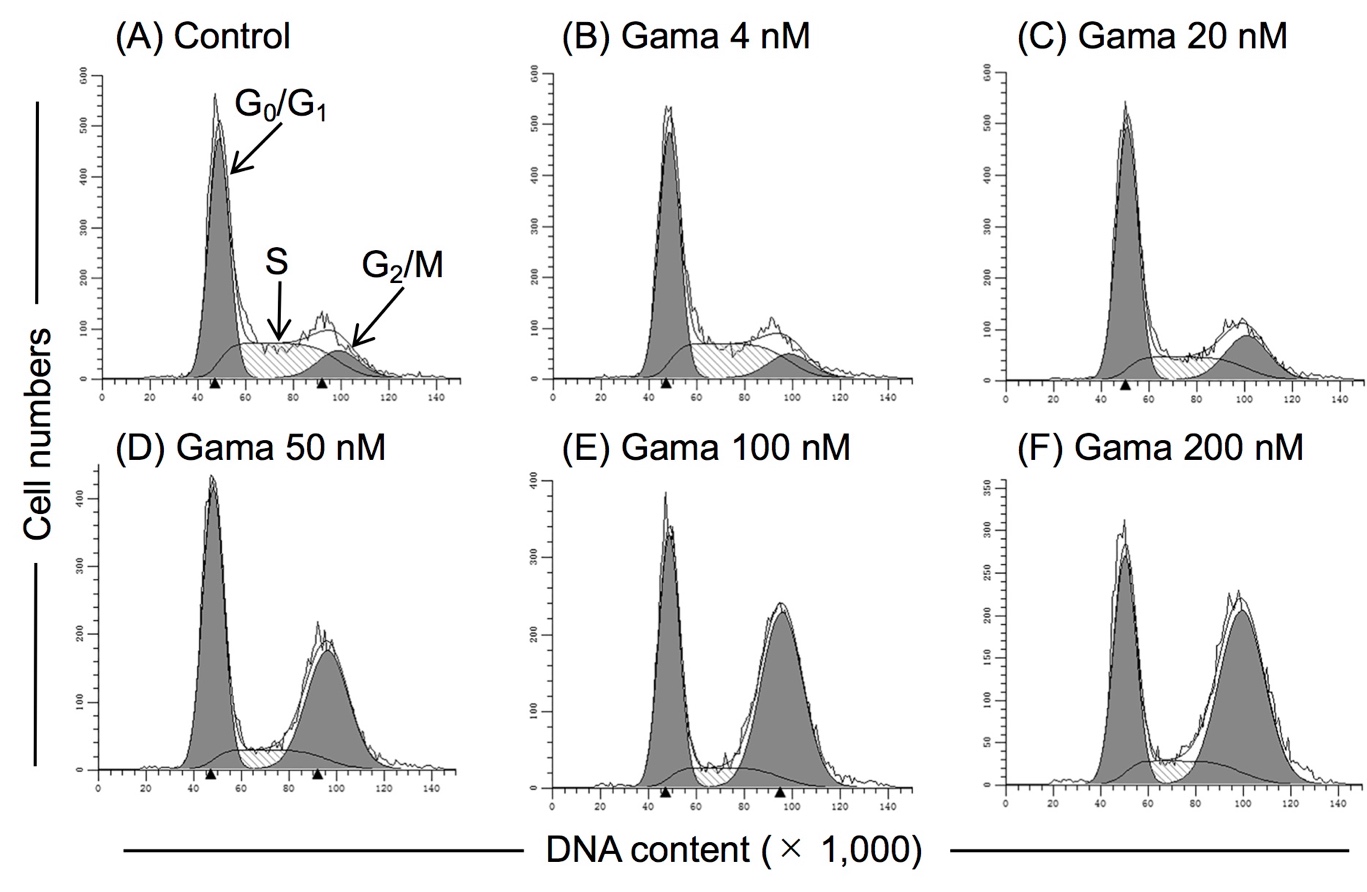


**Supplementary Figure 3.** Effects of gamabufotalin on the cell cycle profiling of U-87 cells**.** Following treatment with various concentrations of gamabufotalin (4, 20, 50, 100 and 200 nM) for 48 h, cell cycle profiling was performed by FACSCanto flow cytometer as described under Materials and methods [(A)-(F)]. Analyzed data and profiles for each G_0_/G_1_ and G_2_/M phase using Diva software and ModFit LT^TM^ ver. 3.0. are shown in the gray area. Cells at S phase are shown as shaded area. A representative FACS histogram from three separate experiments is shown. Gama, gamabufotalin.


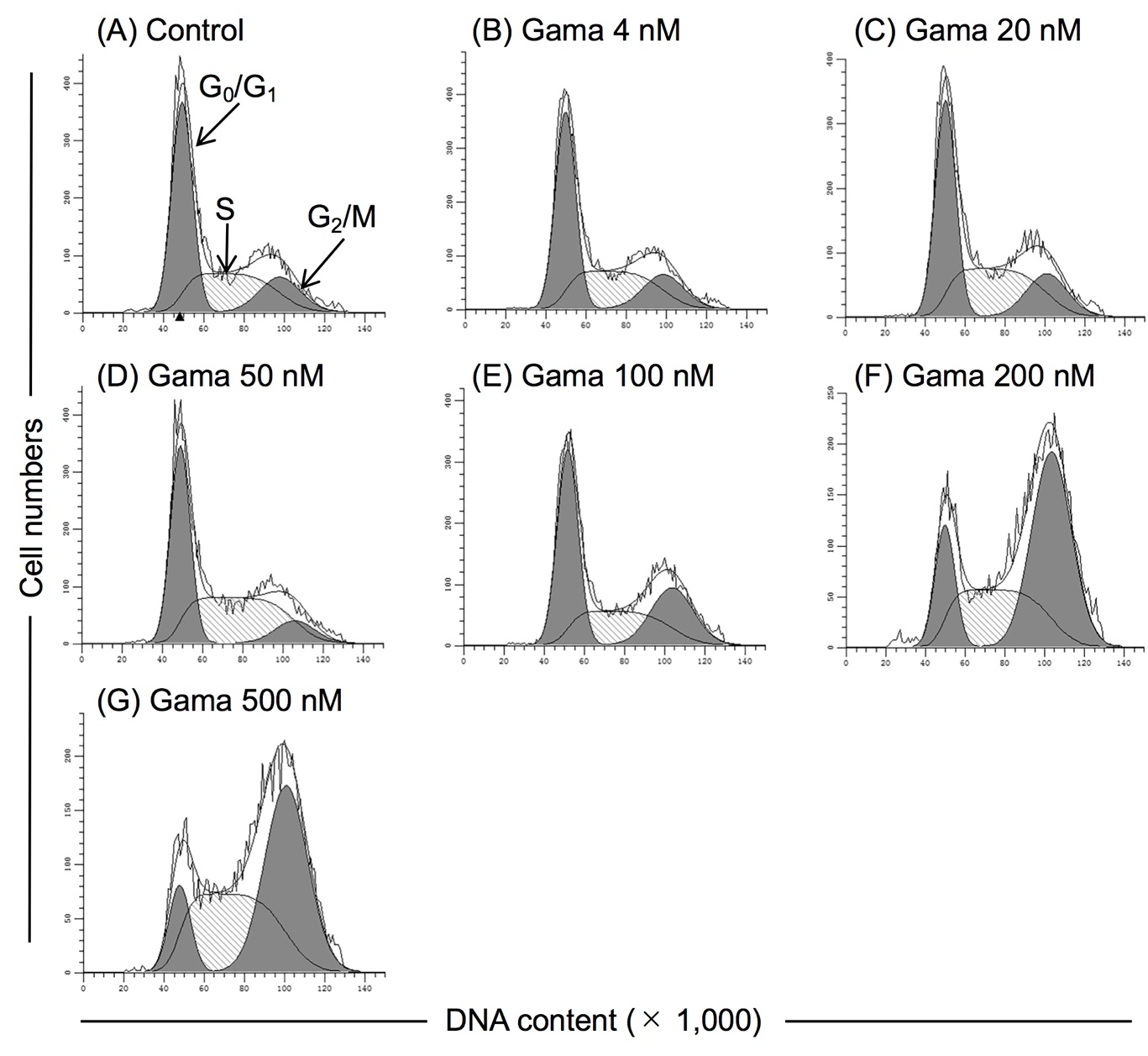


**Supplementary Figure 4.** Effects of gamabufotalin on the cell cycle profiling of U-251 cells**.** Following treatment with various concentrations of gamabufotalin (4, 20, 50, 100, 200 and 500 nM) for 48 h, cell cycle profiling was performed by FACSCanto flow cytometer as described under Materials and methods [(A)-(G)]. Analyzed data and profiles for each G_0_/G_1_ and G_2_/M phase using Diva software and ModFit LT^TM^ ver. 3.0. are shown in the gray area. Cells at S phase are shown as shaded area. A representative FACS histogram from three separate experiments is shown. Gama, gamabufotalin.


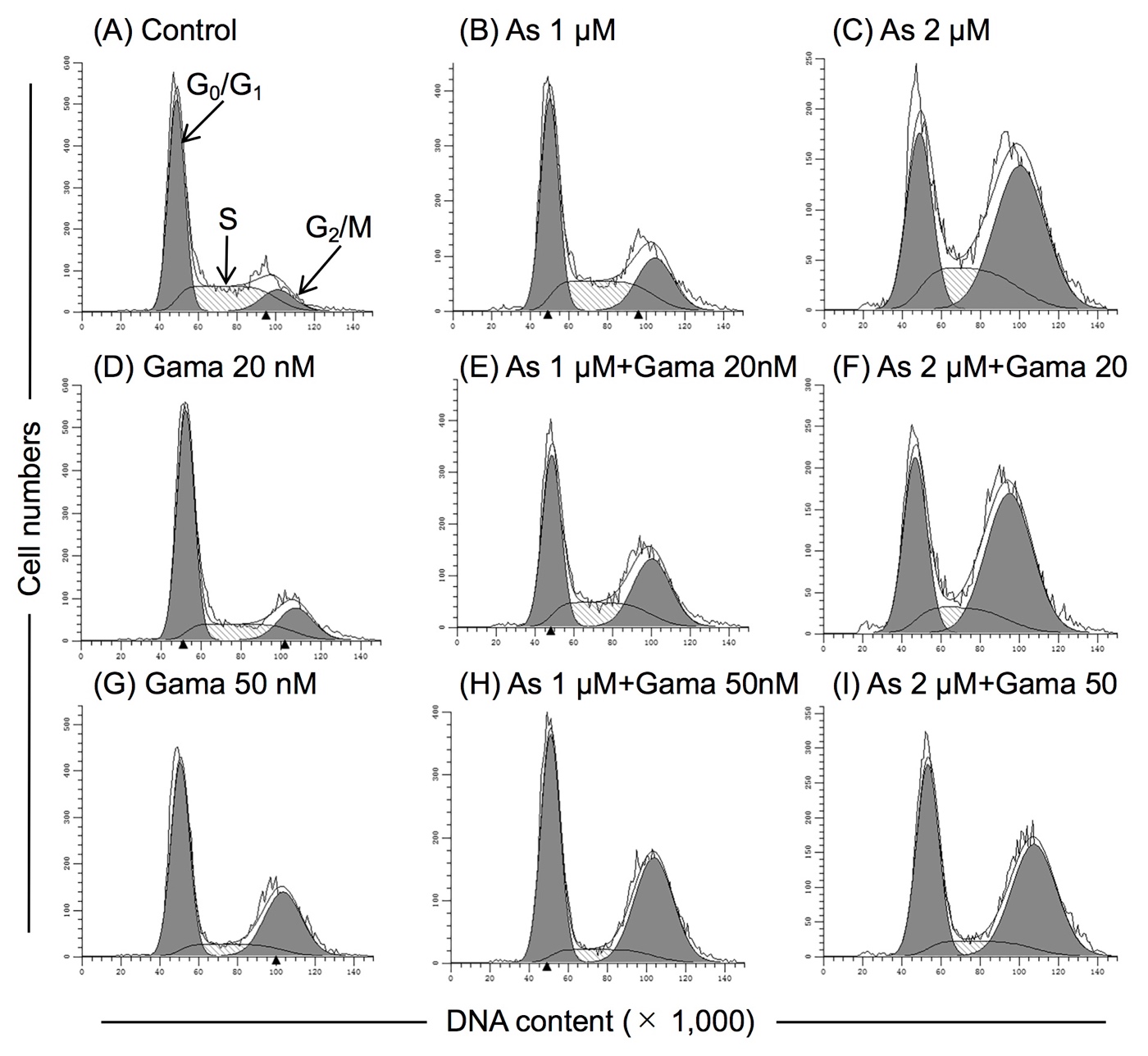


**Supplementary Figure 5.** Effects of relatively low concentrations of As^III^ and gamabufotalin, alone or in combination, on the cell cycle profiling of U-87 cells**.** Following treatment for 48 h with As^III^ (1 and 2 µM) and gamabufotalin (20 and 50 nM), alone or in combination, cell cycle profiling was performed by FACSCanto flow cytometer as described under Materials and methods [(A)-(I)]. Analyzed data and profiles for each G_0_/G_1_ and G_2_/M phase using Diva software and ModFit LT^TM^ ver. 3.0. are shown in the gray area. Cells at S phase are shown as shaded area. A representative FACS histogram from three separate experiments is shown.
